# DriverRWH: discovering cancer driver genes by random walk on a gene mutation hypergraph

**DOI:** 10.1186/s12859-022-04788-7

**Published:** 2022-07-13

**Authors:** Chenye Wang, Junhan Shi, Jiansheng Cai, Yusen Zhang, Xiaoqi Zheng, Naiqian Zhang

**Affiliations:** 1 School of Mathematics and Statistics, Shandong University, Weihai, 264209 China; 2 Department of Mathematics, Weifang University, Weifang, 261061 Shandong China; 3 Department of Mathematics, Shanghai Normal University, Shanghai, 200234 China

**Keywords:** Cancer driver genes, Somatic mutation, Gene network, Hypergraph model, Random walk, Candidate gene prioritization

## Abstract

**Background:**

Recent advances in next-generation sequencing technologies have helped investigators generate massive amounts of cancer genomic data. A critical challenge in cancer genomics is identification of a few cancer driver genes whose mutations cause tumor growth. However, the majority of existing computational approaches underuse the co-occurrence mutation information of the individuals, which are deemed to be important in tumorigenesis and tumor progression, resulting in high rate of false positive.

**Results:**

To make full use of co-mutation information, we present a random walk algorithm referred to as DriverRWH on a weighted gene mutation hypergraph model, using somatic mutation data and molecular interaction network data to prioritize candidate driver genes. Applied to tumor samples of different cancer types from The Cancer Genome Atlas, DriverRWH shows significantly better performance than state-of-art prioritization methods in terms of the area under the curve scores and the cumulative number of known driver genes recovered in top-ranked candidate genes. Besides, DriverRWH discovers several potential drivers, which are enriched in cancer-related pathways. DriverRWH recovers approximately 50% known driver genes in the top 30 ranked candidate genes for more than half of the cancer types. In addition, DriverRWH is also highly robust to perturbations in the mutation data and gene functional network data.

**Conclusion:**

DriverRWH is effective among various cancer types in prioritizes cancer driver genes and provides considerable improvement over other tools with a better balance of precision and sensitivity. It can be a useful tool for detecting potential driver genes and facilitate targeted cancer therapies.

**Supplementary Information:**

The online version contains supplementary material available at 10.1186/s12859-022-04788-7.

## Background

Cancer is a complex genetic disease characterized by abnormal and uncontrolled cellular growth, which is caused primarily by the accumulation of genomic alterations that together enable malignant growth [1, 2]. Recent advances in next-generation sequencing (NGS) technologies have generated massive amounts of cancer genomic data, such as The Cancer Genome Atlas (TCGA), which provides somatic mutation landscapes to better characterize the molecular signatures of cancer [3]. There is a consensus viewpoint on tumorigenesis that only a few mutational events occurring in a set of genes (called “cancer driver genes”) affect the homeostatic development of a set of key cellular functions [4–6]. Discovery of these cancer driver genes across various tumor types is a key step in understanding tumor biology and developing targeted anticancer therapies.

A number of computational tools have been developed to identify cancer driver genes from multidimensional genomic data. Most of these tools can be classified into three categories based on their basic principles [7]. Frequency-based approaches define that the most commonly occurring mutation are more likely to be drivers, such as MutSigCV and MuSic [8, 9]. Unfortunately, methods based on frequency are underpowered for uncovering low recurrently driver genes [10]. Functional impact-based approaches, such as OncodriveFM, integrate multiple-domain information to predict the functional impact of single nucleotide variants (SNVs) [11, 12]. However, most of these methods use machine learning based models. Building either a gold-standard positive data set or a negative data set for such model is a difficult task, and that restricts the use of these methods [10]. The third category is network-based methods enlightened by the observation that mutations in a cancer genome tend to converge on a few biological pathways, attempt to identify groups of driver genes based on prior knowledge of pathways and proteins or genetic interactions [13–17]. A tool named DawnRank adopts PageRank algorithm to rank potential drivers based on their impact on the overall differential expression of the downstream genes [14]. HotNet2 uses a random walk with restart algorithm for identification of mutated subnetworks, in which the mutation frequency of each gene and the frequencies of its network neighbors are considered and hub genes are often yielded with highly predicted scores [15]. This kind of methods have advantages in their ability to identify driver genes with low recurrence and improve the accuracy of predicting driver genes to some extent [18].

Despite the rapid progress in computational approaches to prioritize cancer driver genes with the advent of next-generation sequencing technologies, the false positive rates of these existing methods are still too high. In addition, there are evidences showing that driver gene co-occurring may play a key role in cancer initiation and progression [19–21]. Because the activation or inactivation of one given driver gene is usually not sufficient to induce tumorigenesis, multiple mutations in different driver genes have to cooperate to gradually transform normal cells into precursor lesions and subsequently invasive and metastatic cancer [22–25]. Among majority of the published methods, the practice of putting single gene mutation frequency as input information could result in the loss of all the co-occurring alternations information of the individual tumors. In this study, we introduced a weighted hypergraph model and present a novel tool DriverRWH by integrating mutation profile and PPI network data to predict driver genes. Hypergraph is a generalization of simple graphs where its edges, called hyperedges, are allowed to connect arbitrary number of vertices, which makes it suitable for representation of high-order relations and it can be used to model biology network, data structure, computations and a variety of other systems [26–28]. Herein, we adopted hyperedges to represent the co-exist relationship among mutated genes in individuals, so the problem of information loss of co-occurring alternations can be avoided in a certain extent. We next specified the weights of mutated genes in each hyperedge according their interaction in PPI network and construct the weighted hypergraph. Thereafter, we generalized a random walk algorithm to the weighted hypergraph. Finally, we ranked all the candidate mutated genes for the given cancer type. To verify our method, we applied DriverRWH to 31 cancer types from TCGA and found that our method outperforms the state-of-the-art tools for the majority of cancer types regardless of which reference network we use. We also evaluated the robustness of our method and found that DriverRWH is highly robust to various data perturbations.

## Methods

### Overview

**Fig. 1:**
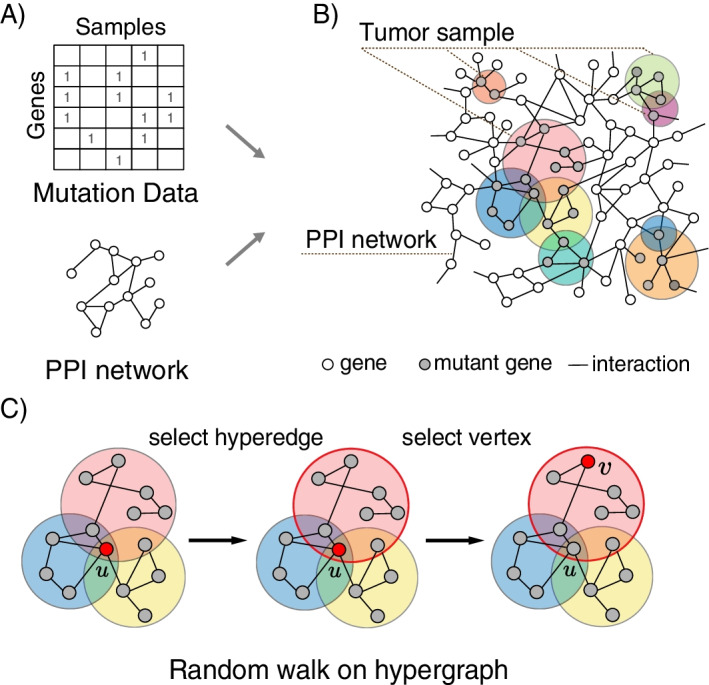
Overview of DriverRWH. **A**, **B** Construction of the weighted hypergraph model using somatic mutation profiles of a given cancer type and a PPI network. Each sample is indicated with colored circular area (hyperedge) which contains all the mutated genes (vertices) of individual. Since the number of mutated gene varies from samples, the hypergraph contains different number of vertices. The weights of vertices in each hyperedge are assigned according to the degree in the context of the background subnetwork. **C** Illustration of the random walk process on the hypergraph. For vertex $u$, we randomly select a hyperedge which incident with $u$ and then selects a node according the weights of vertices in selected hyperedge as the destination vertex $v$ to shift

In this study, we proposed DriverRWH, which uses random walk on weighted hypergraph to prioritize the driver genes (Fig. 1). Firstly, for a given cancer type, a hypergraph was constructed basing on mutation profile, wherein tumor samples are presented as hyperedges and mutant genes are presented as vertices. Secondly, according to our hypothesis that a gene is more likely to be a driver gene if it is highly associated with other mutated genes, we differentiated genes within a hyperedge of sample in accordance with their degrees in the corresponding subnetwork of the PPI network. Then, we adopted a probabilistic weighted random walk that take advantage of the hypergraph structure, and carried out this iteratively. After some steps, the random walk would stabilize, producing a score for each mutated gene. At last, all candidate mutant genes are ranked in descending order based on their score.

### The DriverRWH algorithm

In the present model, mutation data of a given cancer type and a PPI dataset are used as the input information (Fig. 1A). As shown in Fig. 1B, a hypergraph consisting of the mutated genes of all samples was constructed. If a gene is mutated in a sample, it would be presented as a vertex in the hyperedge corresponding to the sample. Without loss of generalization, the hypergraph can be defined as $HG(V,\mathcal{E})$, where $V$ is the set of vertices and $\mathcal{E}$ is the set of hyperedges. A hyperedge $e$ is a subset of, satisfying $\bigcup\limits_{{e \in {\mathcal{E}}}} = V$. Hyperedge $e$ is said to be incident with vertex $u$ if $u\in e$; thus, the incidence matrix $H\in {R}^{|V|\times |\mathcal{E}|}$ can be defined as follows:

$$h\left( {u,e} \right) = \left\{ {\begin{array}{*{20}l} 1 \hfill & {{\text{ if }}u \in e} \hfill \\ 0 \hfill & {{\text{ if }}u \notin e} \hfill \\ \end{array} } \right.$$

After construction of the hypergraph, a specified subnetwork is generated for each sample, based on the mutated genes and their interaction in the PPI network. According to our hypothesis that a gene is more likely to be a driver gene if it is highly associated with other mutated genes, a fairly standard choice of the weight of vertices in each hyperedge are their degrees in the corresponding induced subnetwork of the PPI network.

Then, we developed a random walk process on the weighted hypergraph. Similar to a random walk on a simple graph, this walk is a type of Markov process, which is seen as the transition between two vertices. Note that the transition on the hypergraph occurs only if two vertices are incident to a hyperedge, so the random walk on the hypergraph is defined to be a two-step process. In the first step, the surfer selects a hyperedge $e$ incident with the current vertex $u$; thereafter, it selects a target vertex $v$ within the chosen hyperedge (Fig. 1C). If one vertex is an isolated node in the subnetwork, it also has the potential to be a driver gene, so a small weight of 0.01 is set. Let $Ne$ be the subnetwork containing vertices in hyperedge $e$ and denote $d_{Ne} \left( u \right)$ as the degree of $u$ in the subnetwork.

$$w\left( {u,e} \right) = \left\{ {\begin{array}{*{20}c} {d_{Ne} \left( u \right),} & {{\text{ if }}u \in e} \\ {0.01,} & {{\text{ if }}u \notin e} \\ \end{array} } \right.$$

Thereafter, the surfer selects vertex $v$ proportional to the weight of $v$ within the hyperedge. Notably, in our model, the weights of vertices may vary in accordance with the hyperedges. According to the aforementioned definition, the degree of vertex $u$ and hyperedge $e$ in hypergraph $HG\left( {V,{\mathcal{E}}} \right)$ can be defined as follows:

$$d\left( u \right) = \mathop \sum \limits_{{e \in {\mathcal{E}}}} h\left( {u,e} \right)$$

$${ }\delta \left( e \right) = \mathop \sum \limits_{u \in e} w\left( {u,e} \right)$$

With all the elements defined, we calculated the transition probability from vertex $u$ to vertex $v$ as follows:

$$P\left( {u,v} \right) = \mathop \sum \limits_{{e \in {\mathcal{E}}}} \frac{{h\left( {u,e} \right)}}{d\left( u \right)}\frac{{w\left( {v,e} \right)}}{{\mathop \sum \nolimits_{{\hat{v} \in e}} w\left( {\hat{v},e} \right)}}$$

which can also be written in matrix form:

$$P = D_{u}^{ - 1} HD_{e}^{ - 1} W^{T}$$

where ${D}_{u}\in {R}^{\left|V\right|\times \left|V\right|}$ is the diagonal vertex degree matrix, ${D}_{e}\in {R}^{|\mathcal{E}|\times |\mathcal{E}|}$ is the diagonal hyperedge degree matrix with element $\delta (e)$ and $W\in {R}^{|V|\times |\mathcal{E}|}$ is the weighted incident matrix of hypergraph $HG(V,\mathcal{E})$. Note that the transition matrix $P$ is stochastic, where each row sums to 1.

Furthermore, we implemented a random walk with restart on the hypergraph. All genes are considered to be potential driver genes and are assigned with equal probabilities; i.e., the initially normalized probability vector $\overrightarrow{v}(0)\in {R}^{|V|\times 1}$ such that each element is assigned with equal probability $\frac{1}{\left|V\right|}$. Moreover, the restart probability at every step is set to be $1-\alpha (0<\alpha <1)$. In this article, we set $\alpha$ to be 0.2. Finally, the random walk formula can be expressed as follows:

$$\vec{v}\left( {t + 1} \right) = \alpha P^{T} \vec{v}\left( t \right) + \left( {1 - \alpha } \right)\vec{v}\left( 0 \right){ },{ }t = 0,1,2, \ldots$$

In the formula above, $\overrightarrow{v}(t)$ is defined such that the $i$ th element means the probability that the surfer stops at vertex $i$ at step $t$. After a number of steps, the random walk will be stable, which can be defined as $\overrightarrow{v}(\infty )$. The stabilized state implies that the distance between $\overrightarrow{v}(t+1)$ and $\overrightarrow{v}(t)$ by the L1 norm is smaller than the provided cutoff value. In this paper, we set the cutoff as ${10}^{-6}$. The elements of the stabilized vector $\overrightarrow{v}$ are defined as the DriverRWH score, which can reflect the role that the mutated genes play in cancer.

### Datasets and networks

Somatic mutation data for 9183 tumor samples across 31 cancer types (Additional file 2) used in this work are available from TCGA, which were downloaded by UCSC Browser (https://xenabrowser.net/datapages/) [29]. We downloaded two independently developed PPI datasets from the STRINGv10 (https://string-db.org) [30] and the HumanNet (http://www.functionalnet.org/humannet/) [31].

### Performance evaluation

To evaluate the method, an unbiased comprehensive known cancer gene set is needed. Unfortunately, such a gold-standard set of cancer genes is currently unavailable. Alternatively, we used four complementary cancer gene sets derived from various sources as the reference driver gene set for all the cancer types. First, 616 cancer genes were downloaded from the Cancer Gene Census (CGC) database, which includes genes for which mutations have been causally implicated in cancer and is widely used as a gold-standard cancer gene set [32]. Second, the list of HiConf cancer gene panels consists of 99 driver genes that have previously been detected through genetic criteria and that could plausibly be detected with exome sequencing data [33]. The third set has 291 high-confidence cancer driver genes identified by a rule-based method (HCD) [34]. The fourth set contains 125 driver genes defined by the "20/20 rules", which identifies Mut-driver genes based on the characteristic mutational patterns for oncogenes and tumor suppressor genes [35]. Now that each cancer gene set is biased toward particular features or study methods, we utilized a union of these four lists as the reference driver gene set, with a total of 785 genes. This operation can reduce the bias caused by using a single reference gene list to some degree. Using aforementioned reference driver genes as a benchmark, we generated receiver operating characteristic (ROC) curves and areas under the curve (AUCs) to evaluate the true positive and false positive rate. For practical reasons, only top-ranked candidate genes might enter into follow-up experimental validation. Considering that the high performance of prioritization for all genes cannot guarantee successful prioritization for the top ranked candidates, we also assessed the number of known driver gene recovered in the top 20, 50, 100,150 and 200 candidate genes.

Due to the diversity of cancer types, we are more interested in tumor-specific drivers than the general common drivers across all tumor types. We downloaded IntOGen database (https://www.intogen.org/download) [4]. This database harnesses the strengths of different driver prediction methods and provides a tumor-specific driver genes list, which is considered to be the best trade-off between sensitivity and specificity. This list contains 31 types of cancer among which Kidney Chromophobe (KICH) has 7 specific drivers (minimum) and Uterine Corpus Endometrial Carcinoma (UCEC) has 55 (maximum). All of the above lists are shown in Additional file 3. From an application point of view, we should assess the ability of our method to identify novel driver genes that may not have been discovered in IntOGen. The genes in top 200 candidate gene list predicted by DriverRWH with both HumanNet and STRINGv10 while not in the tumor-specific drivers were considered to be potential novel drivers. From the functional perspective, these genes were evaluated by the biological analysis using DAVID on-line database, CancerGeneNet and iGMDR database [36–39].

We leveraged a literature mining method named CoCiter, which calculates the co-citation significance between predicted driver genes and the keywords cancer type, ‘driver’ and ‘cancer’ to verify the top 30 significant genes [40]. The higher co-citation score implicates the stronger association between the genes and the key terms. Without loss of generality, we compared DriverRWH with 24 driver gene prediction methods across 31 cancer type, some of which identify significant drivers by *P*-value (the genes with FDR adjusted *P*-value < 0.05) and the rest of methods provide the priority scores for candidate driver genes (the top 30 genes are selected as significant drivers). It is acceptable for the reason that the median number of significant genes for other methods in all data sets is 30.

## Results

### Known driver genes have higher degree in the PPI network

**Fig. 2:**
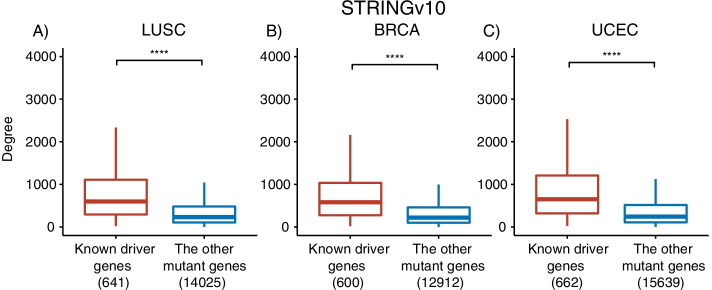
Boxplot comparing the degrees of known driver and the other genes in induced subnetwork. Bracketed digits indicate the number of known driver genes and the other genes in the subnetwork of STRINGv10, which are induced by the mutated genes present in at least one tumor sample for a given cancer type

In DriverRWH, we hypothesized that a gene is more likely to be a cancer driver if it is prone to associate with other mutated genes in cancer. This hypothesis has already been proposed in some studies [15, 41]. To further validate it, we analyzed the linkage of mutated genes in the PPI network. For a given cancer type, an induced subnetwork of the PPI network which just contains mutated genes from all samples was built. The genes that mutated at least once in a cancer type were divided into two groups according to whether they are in the reference driver gene set (the union of CGC, HiConf, MCD, Mut-driver, with a total number of 785 genes): known driver genes and the others. We calculated the degree of vertices in the induced subnetwork. Taking the three cancer types LUSC, BRCA and UCEC for illustration, we found the degrees of known driver genes were significantly larger than those of the other mutant genes (Fig. 2, *P*-value < 0.001). This result suggests that cancer driver genes were adjacent to more mutated genes than the others. The same analysis using HumanNet is also available (Additional file 1: Fig S1).

### Performance of DriverRWH

To evaluate the performance of our method, we compared our method from three aspects, prediction of known driver genes, functional enrichment analysis and literature mining analysis. Firstly, we implemented six prioritizing methods, MutsigCV [8], DawnRank [14], MinNetRank [16], Subdyquency [17], Gravity [41] and OncodriveFML [42] on three cancer types, namely Lung squamous cell carcinoma (LUSC), Breast invasive carcinoma (BRCA), and Uterine Corpus Endometrial Carcinoma (UCEC) (see Additional file 4). In order to eliminate the deviation brought by the background network, we operated DriverRWH and the other three network-based methods (MinNetRank, Subdyquency, and Dawnrank) basing on the same network, STRINGv10 and HumanNet respectively. Then, we compared DriverRWH with 24 other driver gene prediction tools to evaluated its performance across 31 cancer types. Lastly, we verified the robustness of our method by testing the performance in perturbed data where the mutation data and network data were extracted randomly with different size.

#### Results for lung squamous cell carcinoma

Lung cancer is regarded as the main leading cause of cancer deaths, which take up 18.0% of deaths [43]. In this research, we applied DriverRWH to 480 LUSC samples in TCGA database.

**Fig. 3:**
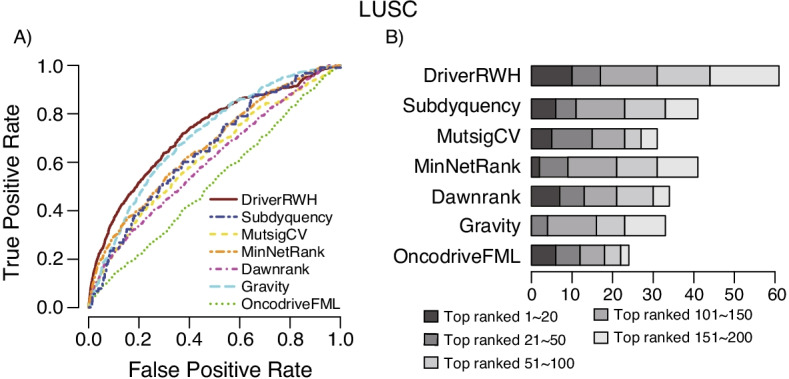
Prediction performance of DriverRWH based on the reference driver set. **A** ROC plots of DriverRWH and other six methods. All the network-based methods, DriverRWH, Subdyquency, MinNetRank and Dawnrank were implemented by using STRINGv10 as background network. **B** Cumulative number of known cancer genes recovered within the top 20, 50, 100, 150 and 200

Using reference driver genes as benchmarks, we generated receiver operating characteristic (ROC) curves. When using STRINGv10 as background network, DriverRWH outperforms the other six tools. in terms of sensitivity and specificity in identifying known driver gene (Fig. 3A). We further assessed the predictive power for the top-ranked candidate genes. As shown in Fig. 3B, we observed that DriverRWH identified more known cancer driver genes by its top 20, 50, 100, 150 and 200 genes. Furthermore, the number of know driver gene retrieved by DriverRWH with STRINGv10 network in its 20 top-ranked candidates is more than half of it. When HumanNet was used, DriverRWH is still significantly better than the others methods (Additional file 1: Fig S2).

To assess the ability of DriverRWH of discovering potential novel cancer driver genes, we considered the genes in the 200 top ranked candidate genes predicted with both HumanNet and the STRINGv10 while not in tumor-specific drivers list, resulting in 72 genes after screening. Biological enrichment analysis using DAVID against Genetic Association Database (GAD) shows that 36 genes (48.6%) are cancer-related (*P*-value = 5.92 × 10^–6^, FDR = 5.92 × 10^–4^) [44]. In particular, these genes are enriched for "lung cancer" (*P*-value = 1 × 10^−3^, FDR = 0.1217). Furthermore, the KEGG pathway enrichment analysis for the potential drivers is encouraging. 8 genes (11.2%) are significantly enriched in pathway: "PI3K - Akt signaling pathway" (*P*-adjust < 0.05), which is significantly related to lung cancer (Additional file 1: Fig S3) [45–48].

Specifically, using the top 30 candidate genes as significant driver, we searched these genes in co-citer website by the key terms ‘Cancer’, ‘Driver’ and ‘Lung’. As Table 1 shows, some significant well-known driver genes like TP53, PTEN and PIK3CA are near the top of the list. Although they are also identified by most of other methods, their ranking fell behind ours. The well-known suppressor TP53 which disrupts the cell cycle arrest and the apoptosis pathways in human cancer ranks first in our method, but it ranks 527th in Gravity algorithm. The PTEN is proved to be related to small cell lung cancer, which is an admitted tumor suppressor gene with phosphatase activity [49]. It is co-cited with ‘Lung’ and ‘Cancer’ for 253 and 2597 times, which is regarded as driver genes in 35 publications. The PTEN ranks the 16th in our list but ranked 44th in MinNetRank and 588th in Gravity. The mutation of PIK3CA gene can lead to abnormal enhancement of the catalytic activity of PI3Ks and promote the carcinogenesis of cells in lung cancer [49]. It ranks 7th in our method but 22th in MutsigCV and OncodriveFML, and 473th in MinNetRank. On the other hand, KDR (Kinase insert domain-containing receptor), ranked 24th, was reported to play a critical role in the metastasis of cancer and is used as a molecular target in cancer therapy [50]. Co-cited with "Cancer" for 207 times and ‘Lung’ for 105 times, KDR even not deemed as a diver gene in lung cancer and can be thought as a potential driver. The similar analysis basing on HumanNet is also available (Additional file 1: Table S1).

**Table 1:** Cociter mining analysis of top 30 LUSC candidate driver genes identified by DriverRWH (STRINGv10)

Genes	Co-appeared count			Is_Specificity	Rank position					
	Lung	Cancer	Driver		MutsigCV	Dawnrank	Gravity	OncodriveFML	Subdyquency	MinNetRank
TP53	854	5942	55	1	1	1	527	3	1	3
TTN	1	8	1	0	2	2771	3959	13,175	NA	1424
DNAH8	0	1	1	0	15	2	NA	2741	3	73
RYR2	3	3	2	0	4	492	400	11,456	2	1706
LRRK2	5	18	1	0	58	3	1556	10,604	NA	223
PTEN	253	2597	35	1	26	6	588	2	14	44
PIK3CA	94	576	13	1	22	36	2536	22	11	473
NOTCH1	84	486	23	1	49	24	1591	20	18	47
CSMD3	0	3	1	0	3	2434	NA	46	NA	940
ANK2	1	4	0	0	99	675	576	13,775	NA	4868
SYNE1	1	2	1	0	11	4	181	4035	NA	8653
KMT2D	1	18	1	1	NA	NA	3147	1	NA	8656
DMD	14	19	3	0	23	419	1998	11,399	NA	566
USH2A	2	4	1	0	6	1136	4591	13,959	NA	215
OBSCN	0	4	0	0	209	2458	204	9252	NA	1487
RYR1	0	3	1	0	53	64	1788	1299	4	590
NF1	12	137	8	1	52	2179	3255	2881	13	1106
LRP1B	8	15	2	0	5	2397	399	9317	NA	1471
APOB	3	23	1	0	84	388	253	8676	NA	1465
RELN	0	9	2	0	113	172	40	13,119	NA	3089
MYH1	1	14	1	0	122	630	NA	6933	NA	2615
EPHA5	2	6	1	0	172	8	NA	7697	28	90
MYH2	4	3	1	0	44	98	NA	5025	7	2538
KDR	105	207	3	0	131	22	549	2832	71	43
HERC2	0	14	1	0	155	5624	148	8436	15	2403
POTEE	1	9	0	0	1153	40	3138	8733	67	229
PIK3CG	36	119	1	0	426	21	602	8186	51	234
CPS1	2	6	1	0	71	5	3852	13,518	NA	387
KMT2C	3	21	4	1	NA	NA	5041	479	NA	4264
HDAC9	5	18	1	0	371	1640	3783	10,894	85	944

We adopted the GAD and KEGG pathway enrichment analysis and found these significant driver genes enrich in the small cell lung cancer, PI3K-Akt signaling pathway, etc., which are significantly related to lung cancer (Additional file 1: Fig S3). The hallmarks of cancer are defined as a set of crucial functional abilities acquired by human cells as they move from normalcy to neoplastic growth states [51]. We linked these significant drivers to hallmarks of cancer using CancerGeneNet online database which calculates the shortest paths between genes and phenotypes [38]. Half of the top 30 genes could be associated with hallmarks of cancer. KDR, one of the potential drivers we mentioned above, is linked to “Angiogenesis”, “Cell Death”, “Differentiation”, “DNA Repair”, “Glycolysis”, “Immortality”, “Inflammation”, “Metastasis” and “Proliferation” (Additional file 5). In order to assess the drug sensitivity of these significant drivers, we performed gene-drug analysis using online database iGMDR, which shows that 73.3% of significant genes are druggable (Additional file 6).

#### Results for breast invasive carcinoma

Breast cancer is the most commonly diagnosed cancer, with an estimate 2.3 million new cases, taking up to 11.7% of all the cancer cases in 2020 [43]. We focused on 791 BRCA samples in TCGA database to construct the hypergraph.

**Fig. 4:**
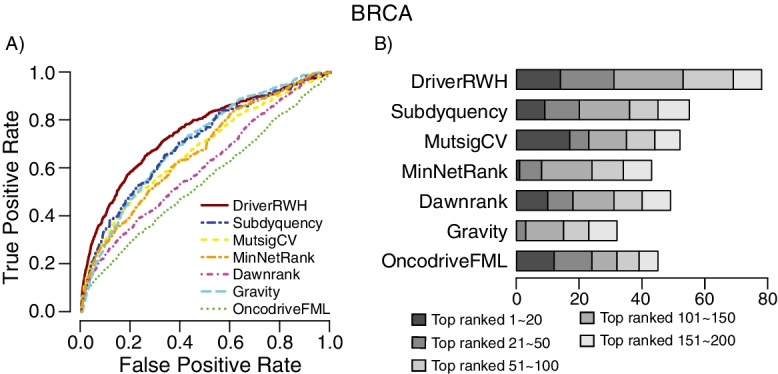
Prediction performance of DriverRWH based on the reference driver set. **A** ROC plots of DriverRWH and other six methods. All the network-based methods, DriverRWH, Subdyquency, MinNetRank and Dawnrank were implemented by using STRINGv10 as background network. **B** Cumulative number of known cancer genes recovered within the top 20, 50, 100, 150 and 200

Compared with other methods, DriverRWH shows the best performance in terms of ROC curves when STRINGv10 and HumanNet were used respectively (Fig. 4 and Additional file 1: Fig S4). Meanwhile, although DriverRWH discerned less driver gene than MutSigCV in top 20 candidates, it was found to predict more known driver genes in the top 50, 100, 150 and 200 candidates (Fig. 4B).

We evaluated the capacity of DriverRWH in identifying the breast cancer potential driver genes. Similarly, we adopted 61 genes, which are in the 200 top ranked candidate genes predicted with both HumanNet and the STRINGv10 while not in tumor-specific drivers list to conduct the GAD and pathway enrichment analysis. Notably, 29 genes (44.6%) are enriched for "CANCER" (*P*-value = 1.67 × 10^–4^, FDR = 1.67 × 10^–4^) and 12 (18.5%) are enriched for "breast cancer" (*P*-value = 2.15 × 10^−5^, FDR = 0.0087). In the case of pathways, these genes are significantly enriched in "Breast cancer". The top 25 pathways are shown in additional file (Additional file 1: Fig S5).

The cociter score of the top 30 candidate genes predicted by DriverRWH using STRINGv10 network is demonstrated in Table 2. Particularly, 8 of the top 10 candidate genes are exactly driver genes, including acknowledged driver gene TP53 (ranked 1st), the most recurrently mutated gene PIK3CA (ranked second), etc. With high cociter scores, KMT2C ranked 8th in DriverRWH, not even identified in MutsigCV and Dawnrank and ranked 2121 in Gravity. AKT1, which co-appears with "Cancer" for 1863 times and "Breast" for 477 times, ranked 10th in DriverRWH while it ranked merely 1226th in Gravity and 2233th in OncodriveFML. The ERBB2, which ranked 16th in DriverRWH, is confirmed to be related to breast cancer, but it ranked 35th in OncodriveFML, 126th in MutsigCV, and even 1465th in Gravity [52]. Besides, DriverRWH can identify some genes that are highly related with breast cancer but was not recognized by other six methods. For instance, EGFR is one of the first identified important targets of novel antitumor agents, which co-occur "Breast" 722 times, "Cancer" 4091 times, and "Driver" 94 times [53]. MTOR ranked 22nd, co-appearing 321 times with "Breast", 1896 times with "Cancer", and 21 times with "Driver". The similar analysis basing on HumanNet is also available (Additional file 1: Table S2).

**Table 2:** Cociter mining analysis of top 30 BRCA candidate driver genes identified by DriverRWH (STRINGv10)

Genes	Co-appeared count			Is_Specificity	Rank position					
	Breast	Cancer	Driver		MutsigCV	Dawnrank	Gravity	OncodriveFML	Subdyquency	MinNetRank
TP53	1177	5942	55	1	1	1	884	5	2	24
PIK3CA	170	576	13	1	2	4	3949	9885	1	200
CDH1	291	1143	13	1	3	2	448	6	4	52
GATA3	84	114	4	1	4	11	179	1	NA	8652
TTN	2	8	1	0	NA	3148	197	16,438	3	6097
PTEN	595	2597	35	1	6	3	300	7	10	42
MAP3K1	59	129	2	1	5	122	208	4	5	528
KMT2C	3	21	4	1	NA	NA	2121	3	NA	4076
DNAH8	0	1	1	0	115	7	NA	1942	78	122
AKT1	477	1863	13	1	NA	6	1226	2233	NA	31
OBSCN	1	4	0	0	NA	1724	642	15,684	14	1760
DMD	1	19	3	0	16	17	1457	5795	11	827
NF1	19	137	8	1	13	998	1641	25	34	926
UBC	176	653	4	0	197	29	NA	2704	38	16
PRDM10	1	1	2	0	916	21	4104	49	560	27
ERBB2	3631	4422	36	1	126	5	1465	35	33	51
MYH9	10	32	4	0	30	358	1540	4141	NA	476
NCOR1	16	58	2	1	9	2903	76	22	7	3160
FOXA1	82	128	5	1	26	26	33	21	NA	8660
ANK3	3	4	2	0	NA	3251	2415	4803	54	2288
LRRK2	4	18	1	0	218	39	7388	3480	278	270
MTOR	321	1896	21	0	NA	22	664	1419	47	115
EGFR	722	4091	94	0	NA	10	2909	14,413	1195	35
RYR2	2	3	2	0	NA	1689	2525	12,929	9	2197
PRKDC	57	274	4	0	77	12	127	9326	51	312
ANK2	0	4	0	0	NA	405	3532	971	44	4349
ASH1L	0	2	1	0	109	5672	957	6540	85	2723
KDM6A	5	24	3	0	64	5042	2140	78	NA	2380
SYNE1	0	2	1	0	NA	1801	1233	12,287	NA	8654
RUNX1	14	110	6	1	7	2058	748	17	28	3175

We performed GAD and pathway enrichment analysis of the top 30 candidate driver genes. The identified genes are enriched in "breast cancer" in GAD. These gene are significantly enriched in "Breast cancer", "Proteoglycans in cancer", "Endometrial cancer", etc., which have an association with breast cancer by KEGG enrichment analysis (Additional file 1: Fig S5). 66.7% of the candidate driver genes could be linked to hallmarks of cancer (Additional file 5). Besides, 86.7% of the identified genes are druggable according to the iGMDR database (Additional file 6).

#### Results for uterine corpus cancer

Uterine corpus cancer is the sixth most common type of cancer and the second most common gynecological malignancy in female, with more than 417,000 new cases and 97,000 deaths worldwide in 2020 [54]. We used 448 patients with 40,543 candidate genes from the TCGA database.

**Fig. 5:**
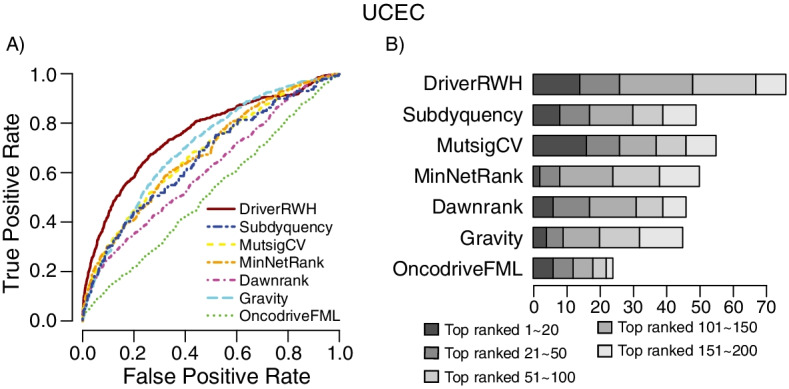
Prediction performance of DriverRWH based on the reference driver set. **A** ROC plots of DriverRWH and other six methods. All the network-based methods, DriverRWH, Subdyquency, MinNetRank and Dawnrank were implemented by using STRINGv10 as background network. **B** Cumulative number of known cancer genes recovered within the top 20, 50, 100, 150 and 200

DriverRWH outperforms the other six prioritizing methods with the same reference driver genes as benchmarks when assessed by the ROC and percentage of known driver gene in the top candidate genes (Fig. 5 and Additional file 1: Fig S6).

For the discovery of potential drives, we selected 41 genes with the same criteria mentioned earlier, of which 22 genes (51.2%) are association with cancer (*P*-value = 1.37 × 10^–4^, FDR = 1.37 × 10^–4^). These genes are significantly enriched in PI3K - Akt signaling pathway and MAPK signaling pathway, both of which play an important role in cellular growth and survival, have been implicated in endometrial cancer pathogenesis (Additional file 1: Fig S7) [55]. 

We took top 30 candidate drivers in consideration, Table 3 shows the cociter score between these candidate genes and the terms " Endometrial", "Cancer" and "Drivers". Apoptosis-suppressing gene MTOR which co-appears with "Endometrial" 63 times, with "Cancer" 1896 times, ranked 19th in DriverRWH, but ranked 112th, 182th, and 1380th in Dawnrank, MutsigCV and OncodriveFML. Notch1 is tumor-suppressive in human endometrial cancer cells [56], which ranked 11th in DriverRWH, while 61th in MutsigCV, 94th in Subdyquency, even 2630th in OncodriveFML and 7054th in Gravity. Moreover, PRKDC is proved to be significantly associated with a high mutation load, which ranked 20th in DriverRWH [57]. Recent research suggest that high mutation load is a predictive biomarker of response to immune checkpoint inhibitors in uterine corpus cancer [58]. The similar analysis basing on HumanNet is also available (Additional file 1: Table S3).

**Table 3:** Cociter mining analysis of top 30 UCEC candidate driver genes identified by DriverRWH (STRINGv10)

Genes	Co-appeared count			Is_Specificity	Rank position					
	Endometrial	Cancer	Driver		MutsigCV	Dawnrank	Gravity	OncodriveFML	Subdyquency	MinNetRank
PTEN	380	2597	35	1	1	1	233	168	1	9
TP53	143	5942	55	1	2	2	1687	403	4	43
PIK3CA	39	576	13	1	3	34	2	673	2	73
CTNNB1	112	2014	29	1	5	5	10	22	11	42
KRAS	51	2538	95	1	4	93	1787	8653	14	218
DNAH8	0	1	1	0	3741	6	6012	NA	24	44
LRRK2	0	18	1	0	2433	13	NA	7114	45	110
OBSCN	0	4	0	0	1171	2641	5199	2055	NA	1184
PRDM10	0	1	2	0	7658	42	NA	992	479	36
RANBP2	0	12	1	0	148	164	40	1176	32	176
NOTCH1	15	486	23	1	61	18	7054	2680	94	45
TAF1	0	9	1	0	45	7954	5227	171	NA	8657
ARID1A	14	67	4	1	31	5994	4505	2	3	1260
ANK3	0	4	2	0	216	5214	5808	157	NA	1914
ATM	4	1222	5	1	241	54	1097	493	16	148
ALB	0	32	1	0	7085	25	7566	7390	397	37
EP300	2	145	2	0	18	151	79	17	54	430
DMD	0	19	3	0	180	69	2776	3448	NA	508
MTOR	63	1896	21	1	182	112	NA	1308	95	63
PRKDC	3	274	4	0	337	40	1217	41	28	130
CTCF	2	50	3	1	1214	29	24	19	NA	164
TTN	0	8	1	0	12	7696	3	1195	NA	4116
FGFR2	23	294	5	1	177	55	4875	1394	43	80
CAD	0	40	1	0	17	27	7174	1091	122	51
NSD1	0	16	2	1	1167	53	34	631	21	85
ASH1L	0	2	1	0	1279	4694	4260	3296	49	2436
TRRAP	0	26	1	0	81	299	8	96	30	303
POTEE	0	9	0	0	4902	155	NA	4873	171	146
GLI3	2	36	2	0	21	20	2263	3088	NA	104
KMT2D	0	18	1	1	23	NA	NA	3113	NA	8652

We performed GAD and pathway enrichment analysis of these candidate genes (Additional file 1: Fig S7). In terms of GAD enrichment analysis, these genes are enriched in "endometrial cancer", etc. In pathway enrichment analysis, they significantly enriched in Endometrial cancer. 70% of the top ranked genes have the shortest path to cancer phenotypes in CancerGeneNet database. PRKDC is linked with “Angiogenesis”, “Cell death”, “Differentiation”, “DNA repair”, “Glycolysis”, “Immortality”, Metastasis” and “Proliferation” (Additional file 5). 83.3% of these candidate genes have related drugs in iGMDR online database (Additional file 6).

#### The stability of the performance across 31 cancer types

**Fig. 6:**
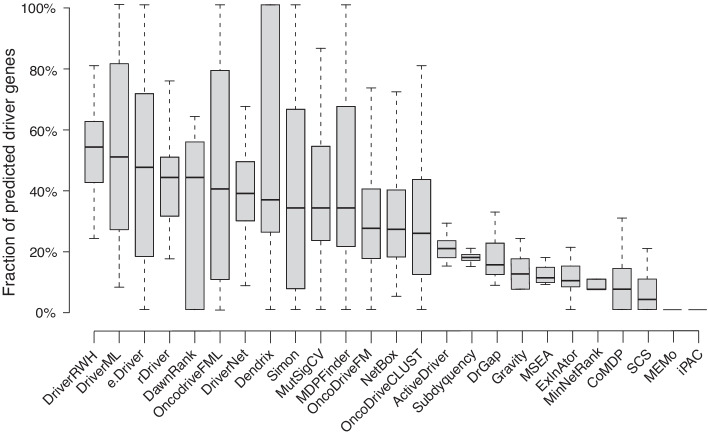
The performance of 25 driver gene prediction methods. Distribution of the fraction of predicted candidate driver genes presented in the reference driver set across 31 cancer types

Furthermore, we compared the performance of DriverRWH with 24 up-to-date driver gene prediction methods in order to assess the stability of DriverRWH across 31 cancer types. For DriverRWH and six methods mentioned above which provide ranks of the candidate driver gene, top 30 genes were selected as significant drivers [59]. For those methods that generate *P*-values, an adjusted *P*-values < 0.05 was used as the threshold to claim driver genes [60, 61]. The details of tools and the criteria for candidate driver genes are provided in the Additional file 7. Figure 6 displays the proportion of predicted driver genes presented in the reference driver set across 31 cancer types, arranged by the order of the median. DriverRWH recovered approximately 50% (median fraction is 53.3%) of known driver genes in the top 30 ranked candidate genes in more than half of 31 cancer types, which is significantly better than the results of the other methods.

#### Robustness of DriverRWH

**Fig. 7:**
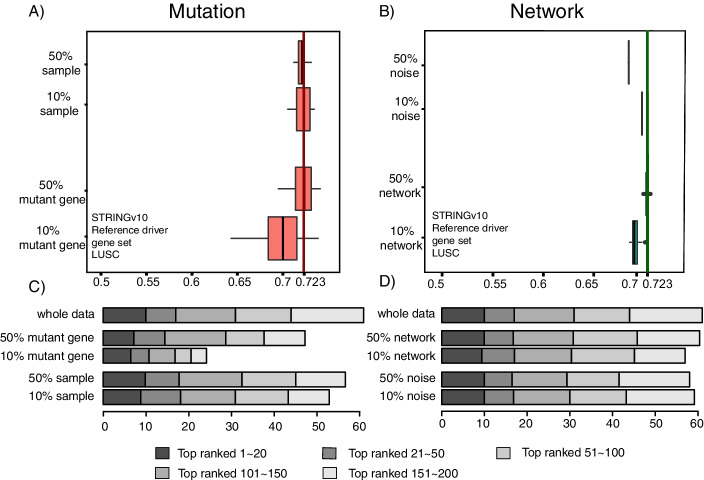
Robustness of DriverRWH. **A**, **B** Boxplots of the effects of different data perturbations on the performance of DriverRWH. The vertical lines represent the AUC scores by DriverRWH using all of the data. **C**, **D** Effects of different data perturbations on the performance of DriverRWH measured by the average cumulative number of known cancer genes recovered in the 20, 50, 100, 150, and 200 top-ranked candidate genes

To test the robustness of DriverRWH, we applied our method to perturbed data where the mutation data and network data were shuffled randomly (Fig. 7). In detail, for the mutation data, two types of perturbations were taken: (1) randomly selecting 50% and 10% of the samples and (2) randomly selecting 50% and 10% of the original mutation information in the somatic mutation matrix. With 20 repeats, we used only 50% and 10% of samples and 50% of mutation information. There is no significant decrease in terms of the AUC scores and the cumulative number of recovered driver genes. If only 10% of mutation information was retained, there would be a slight decrease. It’s worth noting that the performance of the top 20 candidates was always at a high level. For the network data, two forms of perturbation were also taken: (1) randomly selecting 50% and 10% of the original network information and (2) using PPI data with 50% and 10% noise added. There was also only a minor decrease in the AUC scores and the cumulative number of recovered cancer genes. A similar conclusion could be obtained when performing robust analysis basing on HumanNet (Additional file 1: Fig S8). These results suggest that the perturbation of mutation data and the network did not seriously affect the result, indicating that DriverRWH is highly robust to the quality of the input data.

## Discussion

Recent years, many methods have been developed to distinguish driver genes from passengers. Limited by the design of the simple network model, most of them are incapable of expressing the many-to-many multiple association relationship. The mutation profile was always compressed into the mutation frequency of genes, resulting in the loss of co-mutation information for individual samples. In this study, we propose a network-based method DriverRWH, which has the capability of effectively integrating the mutation and PPI network data to predict cancer driver genes. The novelty of our method lies in the introduction of a weighted hypergraph model, which is constructed to simultaneously capture two class of relation among mutated genes in individual samples: 1) high-order relations were captured by storing hundreds of mutated genes in a hyperedge for each sample. 2) using the same mutated genes as above, an induced subnetwork of PPI network can be generated by preserving mutated genes and their interaction in the background network, which represents the pair-wise relations between mutated genes. Our model retains complete co-mutation relations for the mutated genes in individual tumors and these interactions in PPI network, which can adequately embody the implicit inherent peculiarity of them and avoid the loss of information. Taking advantage of hypergraph structure, we extended the typical random walk process on a simple graph to a probabilistic weighted random walk on hypergraph.

Using a reference driver gene set as a benchmark, DriverRWH consistently outperformed the other six state-of-art prioritization methods in terms of the ROC analysis, rank of driver genes and the cumulative number of known driver genes recovered in the top-ranked candidate genes. Moreover, some new unknown potential driver genes which are co-cited by some cancer associated literatures also can be discovered by DriverRWH, meanwhile the high-ranking genes enrich in some significant cancer pathway. At last, taking top 30 as predicted candidate driver genes, we can compare DriverRWH with other non-ranking methods. The results shows that DriverRWH achieves a higher performance than four prioritization methods and 19 other non-ranking methods across 31 cancer types.

Despite of these encouraging results, there are several limitations in the current model. First, for TCGA data, tumor heterogeneity may increase the data bias, and future work should be done to reduce false-positive discoveries by using single-cell genomics data. Second, DriverRWH relies on a broad context molecular network that is still incomplete at present, so refined gene functional networks in the near future could improve the performance of our method. A cancer-specific network might better represent the natural interactions of genes in cancer and potentially provide a more reliable network. Third, our method focuses on general driver gene detection but does not aim to offer personalized means of diagnosis, which is more useful in real applications. In the future, we plan to extend our method to discover drivers in personalized manner.

## Conclusions

Recently, many computational methods and tools have been proposed to identify driver genes. However, long-tail distribution of the mutation frequency of genes in cancer genomes remains a major concern. There are many widely accepted methods based on mutation frequencies, but they fail to comprehensively consider the co-mutation information in individuals. Considering hypergraph has unique advantages of retaining complete co-occurrence information, we introduced the hypergraph theory in driver gene prediction, thus compensating for the co-mutation information loss issue by existing methods. For each hyperedge, degrees of vertex in the corresponding subnetwork of the PPI network were utilized to design the weighted hypergraph, through which we realized the integration of the mutation data and the PPI data. Subsequently, motivated by PageRank algorithm, we implemented the random walk with restart on the hypergraph, and proposed a novel approach DriverRWH to prioritize mutated genes. As demonstrated in this paper, DriverRWH not only excels existing methods in the identification of known driver genes but also is capable of discovering potential driver genes. Furthermore, the model behaves robustly under the perturbation of mutation data and network data. Our results show that DriverRWH can be a useful tool for prioritization driver genes. The source code of DriverRWH is freely available at https://github.com/ShandongUniversityZhanglab/DriverRWH.

## Supplementary Information

**Additional file1**. ** Figure S1**: Boxplot comparing the degrees of known driver and the other genes in induced subnetwork.** Figure S2**: Prediction performance of DriverRWH based on the reference driver set in HumanNet of LUSC.** Figure S3**: The KEGG pathway enrichment analysis for the candidate driver genes of LUSC.** Figure S4**: Prediction performance of DriverRWH based on the reference driver set in HumanNet of BRCA.** Figure S5**: The KEGG pathway enrichment analysis for the candidate driver genes of BRCA.** Figure S6**: Prediction performance of DriverRWH based on the reference driver set in HumanNet of UCEC.** Figure S7**: The KEGG pathway enrichment analysis for the candidate driver genes of UCEC.** Figure S8**: Robustness of DriverRWH in HumanNet.** Table S1**: Cociter mining analysis of top 30 LUSC candidate driver genes identified by DriverRWH (HumanNet).** Table S2**: Cociter mining analysis of top 30 BRCA candidate driver genes identified by DriverRWH (HumanNet).** Table S3**: Cociter mining analysis of top 30 UCEC candidate driver genes identified by DriverRWH (HumanNet).

**Additional file2**. The details for 31 cancer types used in this work.

**Additional file3**. The known driver gene lists used in this work.

**Additional file4**. The top 200 candidate driver genes predicted by DriverRWH, MutsigCV, Gravity, OncodriveFML, Dawnrank, Subdyquency, : and MinNetRank for three cancer types (LUSC, BRCA, UCEC).

**Additional file5**. The linkages between significant drivers and hallmarks of cancer using CancerGeneNet online database

**Additional file6**. The gene-drug analysis results of significant drivers using iGMDR online database

**Additional file7**. The details of 24 tools and the criteria for candidate driver genes.

## Data Availability

The source code and example datasets used in this research can be download form https://github.com/ShandongUniversityZhanglab/DriverRWH

## Abbreviations

TCGA: The cancer genome atlas
NGS: Next-generation sequencing
SNV: Single nucleotide variants
BRCA: Breast invasive carcinoma
LUSC: Lung squamous cell carcinoma
UCEC: Uterine corpus endometrial carcinoma
ROC: Receiver operating characteristic
AUC: Area under curve
PPI: Protein to protein interaction network
CGC: Cancer gene census
HCD: High confidence cancer driver genes
GAD: Genetic association database
