# Supplementary material for: DriverRWH: discovering cancer driver genes by random walk on a gene mutation hypergraph
Source: BMC Bioinformatics. 2022 Jul 13;23:277. doi: 10.1186/s12859-022-04788-7 (PMC9281118; doi:10.1186/s12859-022-04788-7)
Supplement: Supplementary file 1 — Additional file1. Figure S1: Boxplot comparing the degrees of known driver and the other genes in induced subnetwork. Figure S2: Prediction performance of DriverRWH based on the reference driver set in HumanNet of LUSC. Figure S3: The KEGG pathway enrichment analysis for the candidate driver genes of LUSC. Figure S4: Prediction performance of DriverRWH based on the reference driver set in HumanNet of BRCA. Figure S5: The KEGG pathway enrichment analysis for the candidate driver genes of BRCA. Figure S6: Prediction performance of DriverRWH based on the reference driver set in HumanNet of UCEC. Figure S7: The KEGG pathway enrichment analysis for the candidate driver genes of UCEC. Figure S8: Robustness of DriverRWH in HumanNet. Table S1: Cociter mining analysis of top 30 LUSC candidate driver genes identified by DriverRWH (HumanNet). Table S2: Cociter mining analysis of top 30 BRCA candidate driver genes identified by DriverRWH (HumanNet). Table S3: Cociter mining analysis of top 30 UCEC candidate driver genes identified by DriverRWH (HumanNet). [file 12859_2022_4788_MOESM1_ESM.docx]

**Additional file 1: Supplementary document containing additional results basing on HumanNet and biological enrichment analysis.**

DriverRWH: discovering cancer driver genes by random walk on a gene mutation hypergraph

Chenye Wang^1,^^†^, Junhan Shi^1,†^, Jiansheng Cai^2, †^, Yusen Zhang^1^, Xiaoqi Zheng^3^, Naiqian Zhang^1,*^

^1^School of Mathematics and Statistics, Shandong University, Weihai 264209, China. ^2^Department of mathematics, Weifang University, Weifang, Shandong 261061, China. ^3^Department of mathematics, Shanghai Normal University, Shanghai 200234, China.

*To whom correspondence should be addressed.

^+^The authors wish it to be known that, in their opinion, the first three authors should be regarded as Joint First Authors.

Email address:

CW: [w18669341630@163.com](mailto:w18669341630@163.com)

JS: [jshmshijh@163.com](mailto:jshmshijh@163.com)

JC: healthcai@163.com

YZ: zhangys@sdu.edu.cn

XZ: xqzheng@shnu.edu.cn

NZ: [nqzhang@email.sdu.edu.cn](mailto:nqzhang@email.sdu.edu.cn)

**Contents**

Fig S1: Boxplot comparing the degrees of known driver and the other genes in induced subnetwork.

Fig S2: Prediction performance of DriverRWH based on the reference driver set in HumanNet of LUSC.

Fig S3: The KEGG pathway enrichment analysis for the candidate driver genes of LUSC.

Table S1: Cociter mining analysis of top 30 LUSC candidate driver genes identified by DriverRWH (HumanNet).

Fig S4: Prediction performance of DriverRWH based on the reference driver set in HumanNet of BRCA.

Fig S5: The KEGG pathway enrichment analysis for the candidate driver genes of BRCA.

Table S2: Cociter mining analysis of top 30 BRCA candidate driver genes identified by DriverRWH (HumanNet).

Fig S6: Prediction performance of DriverRWH based on the reference driver set in HumanNet of UCEC.

Fig S7: The KEGG pathway enrichment analysis for the candidate driver genes of UCEC.

Table S3: Cociter mining analysis of top 30 UCEC candidate driver genes identified by DriverRWH (HumanNet).

Fig S8: Robustness of DriverRWH in HumanNet.


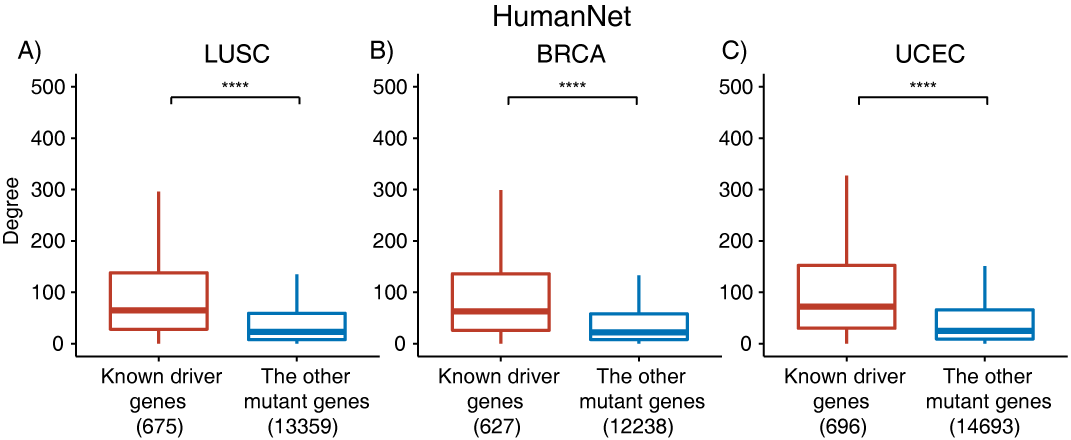


**Fig S1. Boxplot comparing the degrees of known driver and the other genes in induced subnetwork.** Bracketed digits indicate the number of known driver genes and the other genes in the subnetwork of HumanNet, which are induced by the mutated genes present in at least one tumor sample for a given cancer type.


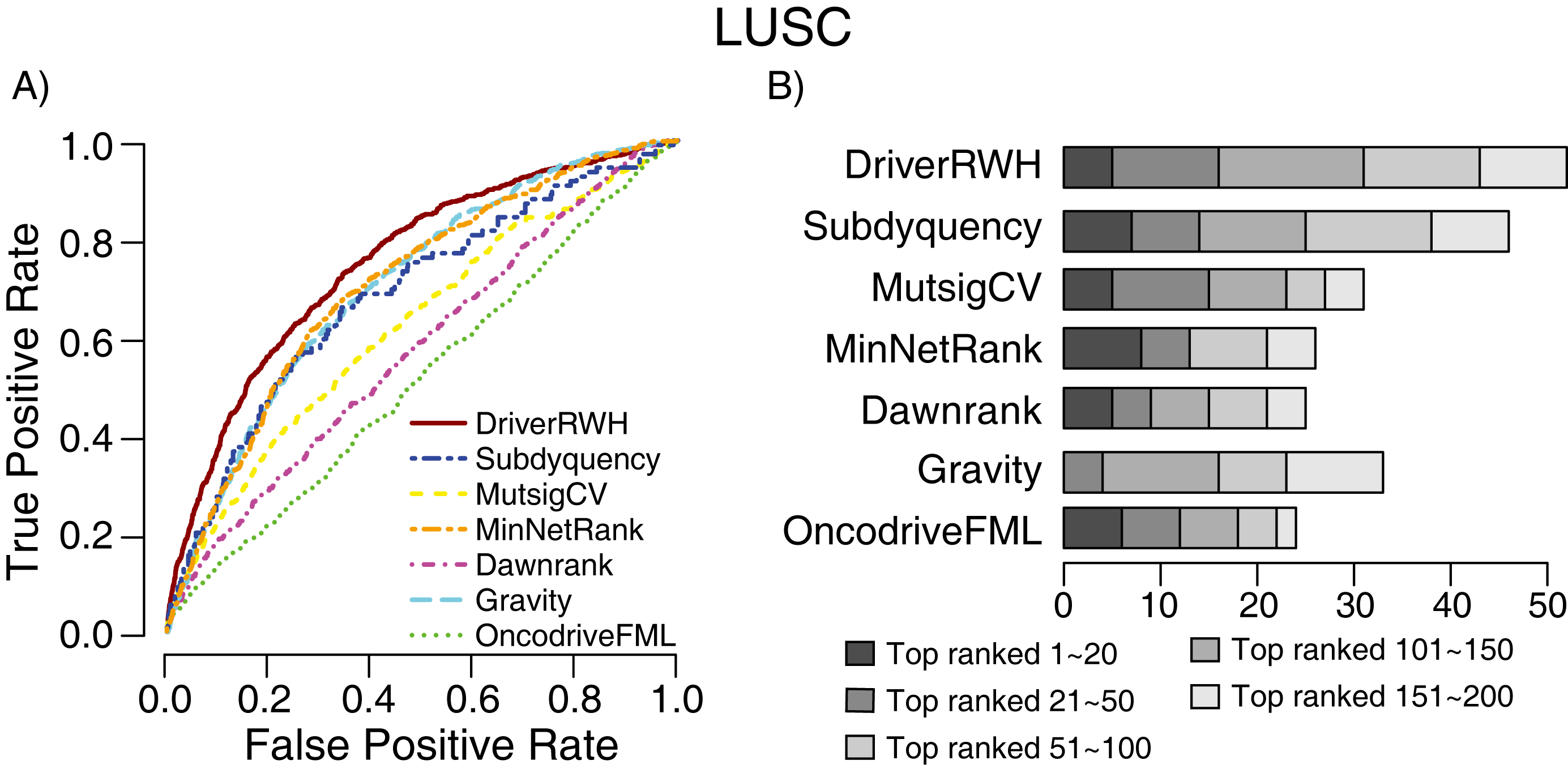


**Fig S2.** **Prediction performance of DriverRWH based on the reference driver set in HumanNet of LUSC.** A) ROC plots of DriverRWH and other six methods. All the network-based methods, DriverRWH, Subdyquency, MinNetRank and Dawnrank were implemented by using HumanNet as background network. B) Cumulative number of known cancer genes recovered within the top 20, 50, 100, 150 and 200.


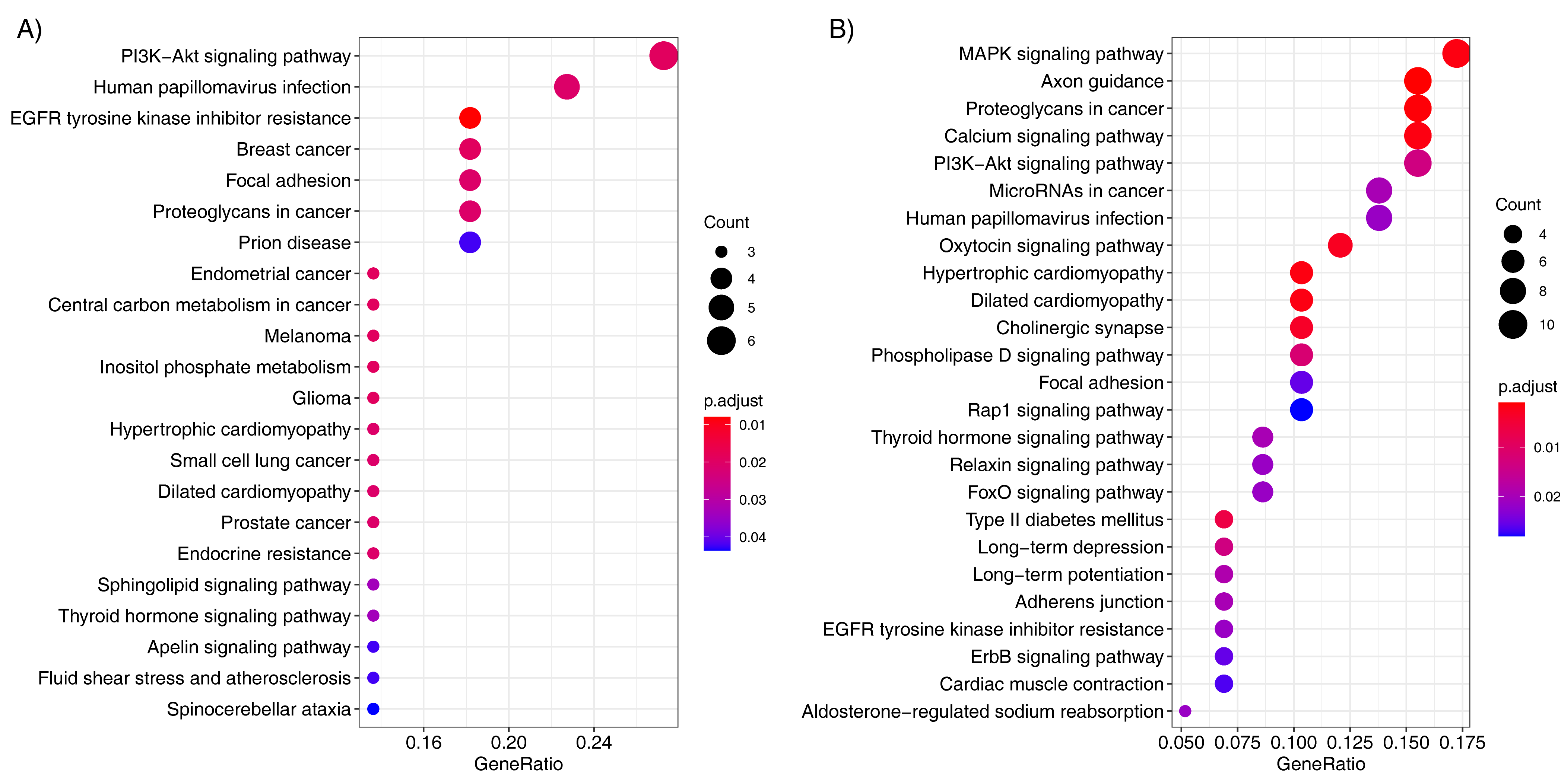


**Fig S3. The KEGG pathway enrichment analysis for the candidate driver genes of LUSC.** The x-axis represents gene ratio. The size of dot represents gene count. The color of the dot represents adjusted p-value. A) The results of top 30 candidate genes. B) The results of potential genes (genes in top 200 candidate gene list predicted by DriverRWH with both HumanNet and the STRINGv10 while not in tumor-specific drivers).

| Genes | Co-appeared count | | | Is_Specificity | Rank position | | | | | |
| --- | --- | --- | --- | --- | --- | --- | --- | --- | --- | --- |
|  | Lung | Cancer | Driver |  | MutsigCV | Dawnrank | Gravity | OncodriveFML | Subdyquency | MinNetRank |
| TP53 | 854 | 5942 | 55 | 1 | 1 | 1667 | 527 | 3 | 1 | 1 |
| TTN | 1 | 8 | 1 | 0 | 2 | 302 | 3959 | 13175 | NA | 29 |
| RYR2 | 3 | 3 | 2 | 0 | 4 | 1 | 400 | 11456 | 2 | 84 |
| KMT2D | 1 | 18 | 1 | 1 | NA | NA | 3147 | 1 | NA | 3121 |
| CACNA1E | 0 | 1 | 1 | 0 | 199 | 4204 | 7215 | 8846 | NA | 3297 |
| ANK2 | 1 | 4 | 0 | 0 | 99 | 694 | 576 | 13775 | NA | 612 |
| MYH2 | 4 | 3 | 1 | 0 | 44 | 74 | NA | 5025 | NA | 3124 |
| RIMS1 | 0 | 1 | 2 | 0 | 365 | 729 | 2996 | 5457 | 17 | 1880 |
| LRP2 | 8 | 9 | 1 | 0 | 36 | 66 | 3331 | 11726 | 8 | 2266 |
| PTEN | 253 | 2597 | 35 | 1 | 26 | 27 | 588 | 2 | 10 | 382 |
| MYH1 | 1 | 14 | 1 | 0 | 122 | 166 | NA | 6933 | NA | 3254 |
| UNC5D | 1 | 4 | 1 | 0 | 241 | 2377 | NA | 5595 | NA | 3426 |
| CACNA1A | 4 | 3 | 1 | 0 | 416 | 1245 | 3697 | 1125 | NA | 75 |
| CREBBP | 14 | 95 | 2 | 0 | 287 | 3841 | 322 | 1904 | NA | 3227 |
| MYH8 | 0 | 0 | 1 | 0 | 80 | 12423 | NA | 1188 | NA | 3314 |
| KALRN | 0 | 5 | 1 | 0 | 243 | 5659 | 3641 | 12179 | 22 | 1307 |
| MAGI2 | 1 | 5 | 1 | 0 | 518 | 5997 | 4463 | 2349 | NA | 3957 |
| MYH4 | 2 | 1 | 1 | 0 | 89 | 4956 | NA | 12989 | NA | 3123 |
| MYH7 | 20 | 29 | 2 | 0 | 225 | 1782 | NA | 2421 | 14 | 252 |
| DLG2 | 0 | 0 | 1 | 0 | 383 | 3421 | 166 | 3173 | NA | 4320 |
| APOB | 3 | 23 | 1 | 0 | 84 | 2 | 253 | 8676 | 6 | 1753 |
| ERBB4 | 25 | 96 | 3 | 0 | 395 | 20 | 2332 | 12240 | NA | 1826 |
| COL1A2 | 18 | 23 | 1 | 0 | 497 | 11 | 32 | 7171 | 25 | 1173 |
| DCC | 19 | 330 | 1 | 0 | 343 | 260 | NA | 7335 | 16 | 111 |
| RIMS2 | 0 | 1 | 1 | 0 | 72 | 319 | 3882 | 6545 | NA | 3455 |
| MAGI1 | 0 | 7 | 2 | 0 | 1185 | 2040 | 1464 | 10077 | NA | 4639 |
| SMARCA4 | 26 | 123 | 5 | 0 | 678 | 104 | 213 | 1094 | 68 | 300 |
| FN1 | 58 | 80 | 2 | 1 | 257 | 2291 | 26 | 2022 | 24 | 3416 |
| COL6A3 | 2 | 5 | 1 | 0 | 427 | 5784 | 37 | 10571 | 18 | 1749 |
| VCAN | 8 | 30 | 1 | 0 | 290 | 4838 | 297 | 8758 | 15 | 3127 |

**Table S1. Cociter mining analysis of top 30 LUSC candidate driver genes identified by DriverRWH (HumanNet).**


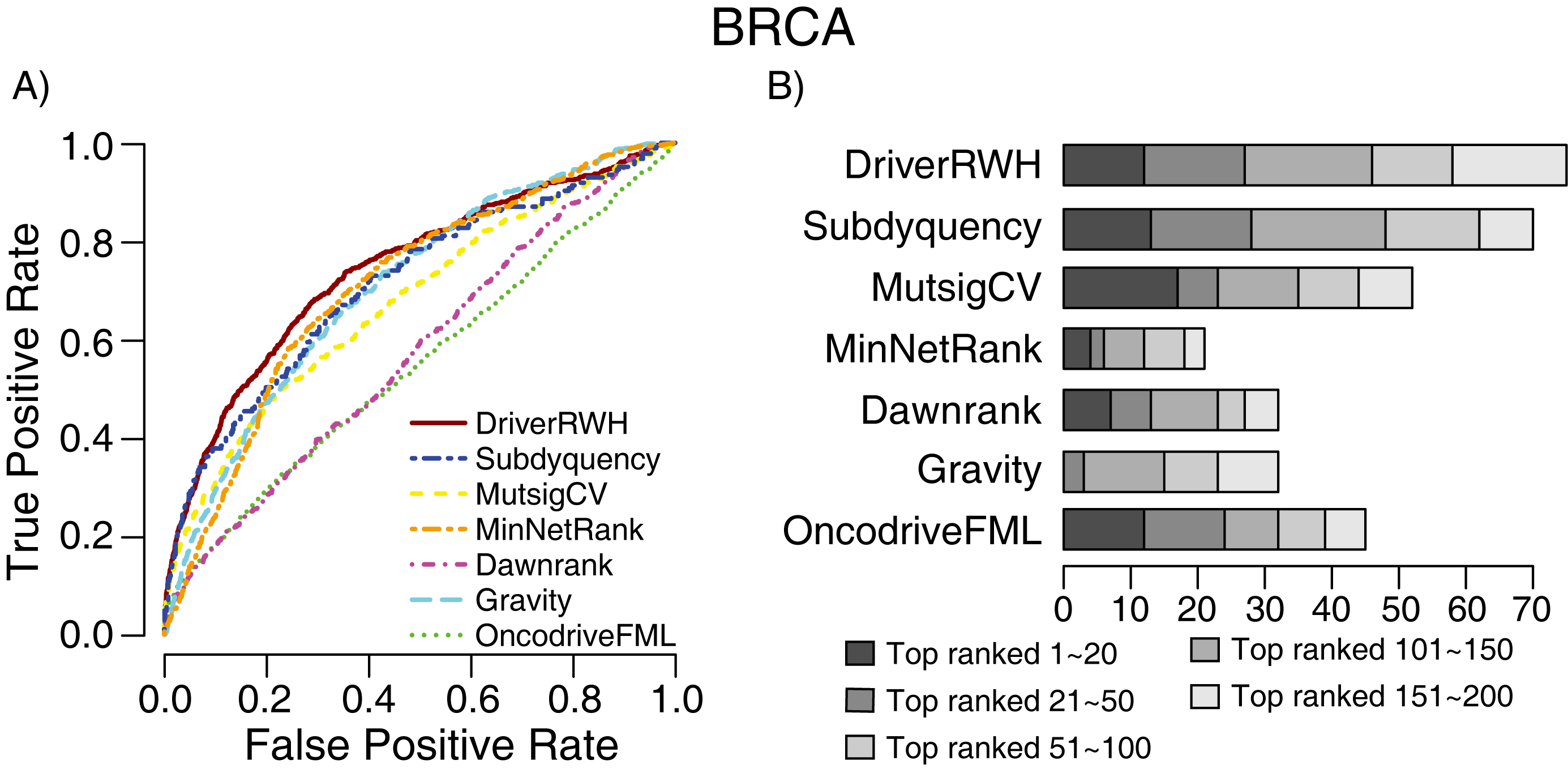


**Fig S4. Prediction performance of DriverRWH based on the reference driver set in HumanNet of BRCA.** A) ROC plots of DriverRWH and other six methods. All the network-based methods, DriverRWH, Subdyquency, MinNetRank and Dawnrank were implemented by using HumanNet as background network. B) Cumulative number of known cancer genes recovered within the top 20, 50, 100, 150 and 200.


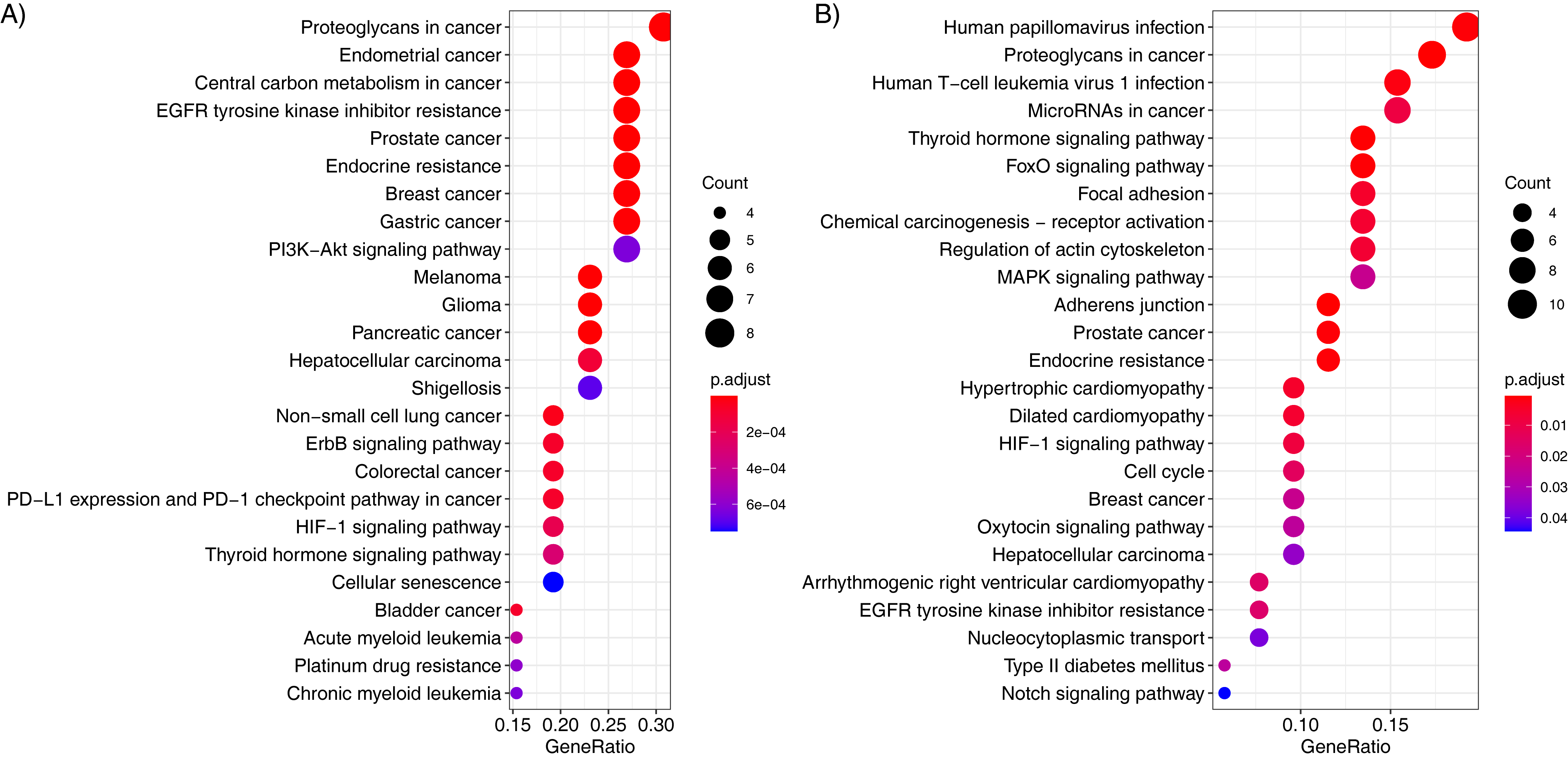


**Fig S5. The KEGG pathway enrichment analysis for the candidate driver genes of BRCA.** The x-axis represents gene ratio. The size of dot represents gene count. The color of the dot represents adjusted p-value. A) The results of top 30 candidate genes. B) The results of potential genes (genes in top 200 candidate gene list predicted by DriverRWH with both HumanNet and the STRINGv10 while not in tumor-specific drivers).

| Genes | Co-appeared count | | | Is_Specificity | Rank position | | | | | |
| --- | --- | --- | --- | --- | --- | --- | --- | --- | --- | --- |
|  | Breast | Cancer | Driver |  | MutsigCV | Dawnrank | Gravity | OncodriveFML | Subdyquency | MinNetRank |
| TP53 | 1177 | 5942 | 55 | 1 | 1 | 2675 | 884 | 5 | 2 | 331 |
| PIK3CA | 170 | 576 | 13 | 1 | 2 | 24 | 3949 | 9885 | 1 | 114 |
| TTN | 2 | 8 | 1 | 0 | NA | 1514 | 197 | 16438 | 3 | 1842 |
| PTEN | 595 | 2597 | 35 | 1 | 6 | 5 | 300 | 7 | 4 | 224 |
| CDH1 | 291 | 1143 | 13 | 1 | 3 | 1409 | 448 | 6 | NA | 2605 |
| GATA3 | 84 | 114 | 4 | 1 | 4 | 3 | 179 | 1 | NA | 3091 |
| AKT1 | 477 | 1863 | 13 | 1 | NA | 11 | 1226 | 2233 | NA | 226 |
| ERBB2 | 3631 | 4422 | 36 | 1 | 126 | 276 | 1465 | 35 | 40 | 1654 |
| CACNA1A | 2 | 3 | 1 | 0 | 142 | 4245 | 3094 | 1208 | 70 | 1440 |
| MAGI1 | 1 | 7 | 2 | 0 | NA | 1595 | 2688 | 4197 | NA | 3587 |
| CREBBP | 38 | 95 | 2 | 1 | 278 | 3753 | 358 | 98 | 90 | 3136 |
| EGFR | 722 | 4091 | 94 | 0 | NA | 9 | 2909 | 14413 | 746 | 465 |
| MAGI2 | 0 | 5 | 1 | 0 | 1377 | 1847 | 3605 | 1083 | NA | 3959 |
| CACNA1E | 0 | 1 | 1 | 0 | 47 | 2731 | 9511 | 13711 | NA | 3229 |
| BRCA1 | 3565 | 4068 | 11 | 1 | 151 | 307 | 480 | 84 | 45 | 112 |
| RB1 | 116 | 604 | 7 | 1 | 19 | 286 | 168 | 18 | 41 | 581 |
| APOB | 1 | 23 | 1 | 0 | 39 | 1 | 4324 | 13312 | 21 | 2934 |
| SMARCA4 | 25 | 123 | 5 | 0 | 542 | 29 | 441 | 4502 | 89 | 324 |
| LRP2 | 0 | 9 | 1 | 0 | 43 | 120 | 2208 | 10621 | NA | 3024 |
| DLG1 | 2 | 52 | 6 | 0 | 128 | 3717 | 2056 | 9996 | NA | 2616 |
| RYR2 | 2 | 3 | 2 | 0 | NA | 10 | 2525 | 12929 | 12 | 1685 |
| SPEN | 1 | 8 | 1 | 0 | 12 | 7396 | 66 | 19 | NA | 3216 |
| KMT2D | 7 | 18 | 1 | 0 | NA | NA | 3832 | 31 | NA | 3230 |
| PRKDC | 57 | 274 | 4 | 0 | 77 | 5480 | 127 | 9326 | 54 | 1373 |
| PPM1E | 0 | 0 | 1 | 0 | 3077 | 3138 | NA | 631 | NA | 5065 |
| CACNA1B | 0 | 0 | 1 | 0 | 149 | 7238 | 8742 | 4887 | NA | 3502 |
| AHCTF1 | 0 | 0 | 2 | 0 | NA | 5537 | 379 | 11064 | NA | 3233 |
| MYH9 | 10 | 32 | 4 | 0 | 30 | 7912 | 1540 | 4141 | 47 | 1802 |
| HUWE1 | 4 | 22 | 2 | 0 | 28 | 8991 | 204 | 10178 | NA | 3181 |
| CASK | 0 | 5 | 1 | 0 | 475 | 956 | 3173 | 7642 | 206 | 486 |

**Table S2. Cociter mining analysis of top 30 BRCA candidate driver genes identified by DriverRWH (HumanNet).**


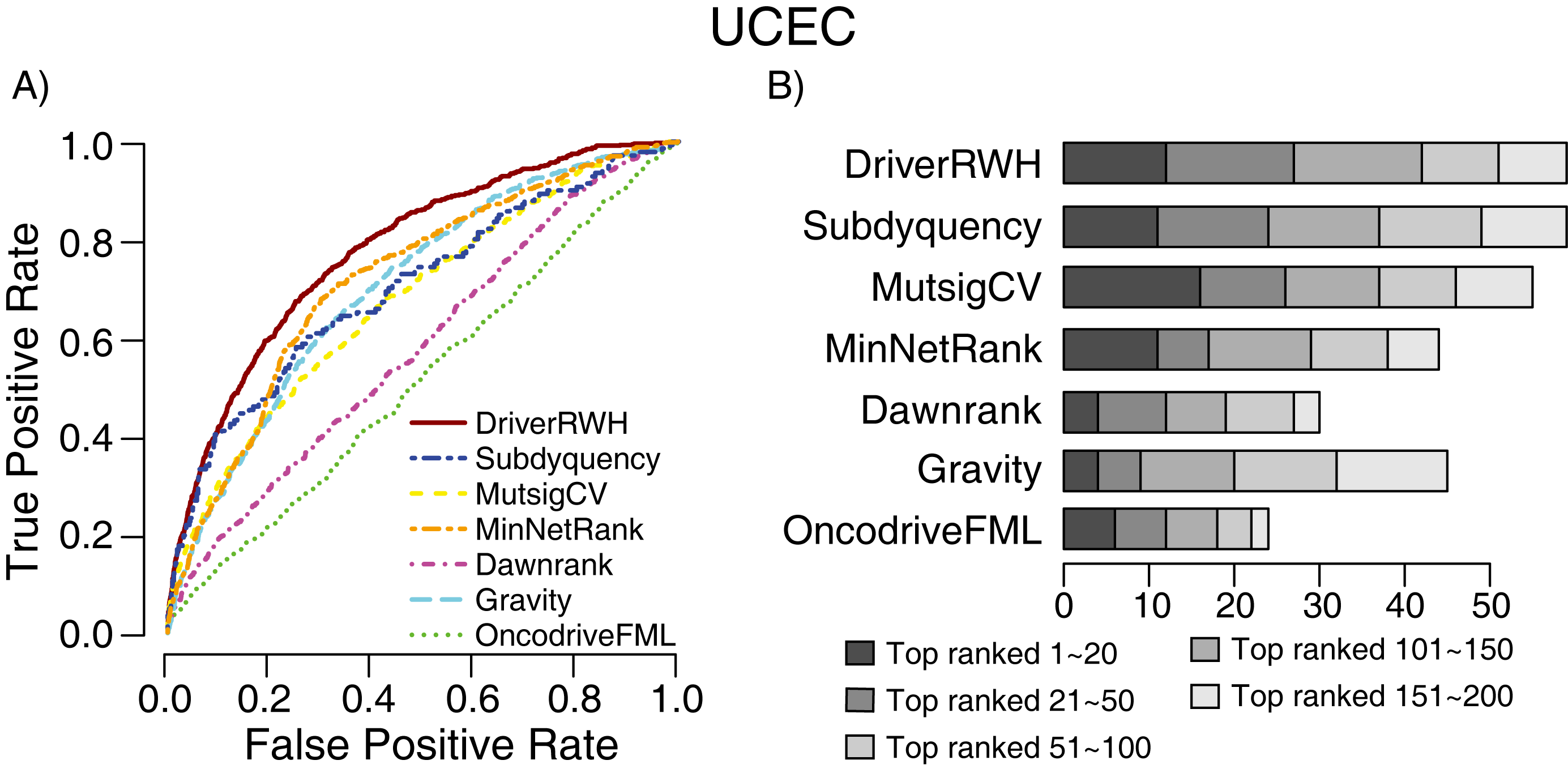


**Fig S6. Prediction performance of DriverRWH based on the reference driver set in HumanNet of UCEC.** A) ROC plots of DriverRWH and other six methods. All the network-based methods, DriverRWH, Subdyquency, MinNetRank and Dawnrank were implemented by using HumanNet as background network. B) Cumulative number of known cancer genes recovered within the top 20, 50, 100, 150 and 200.


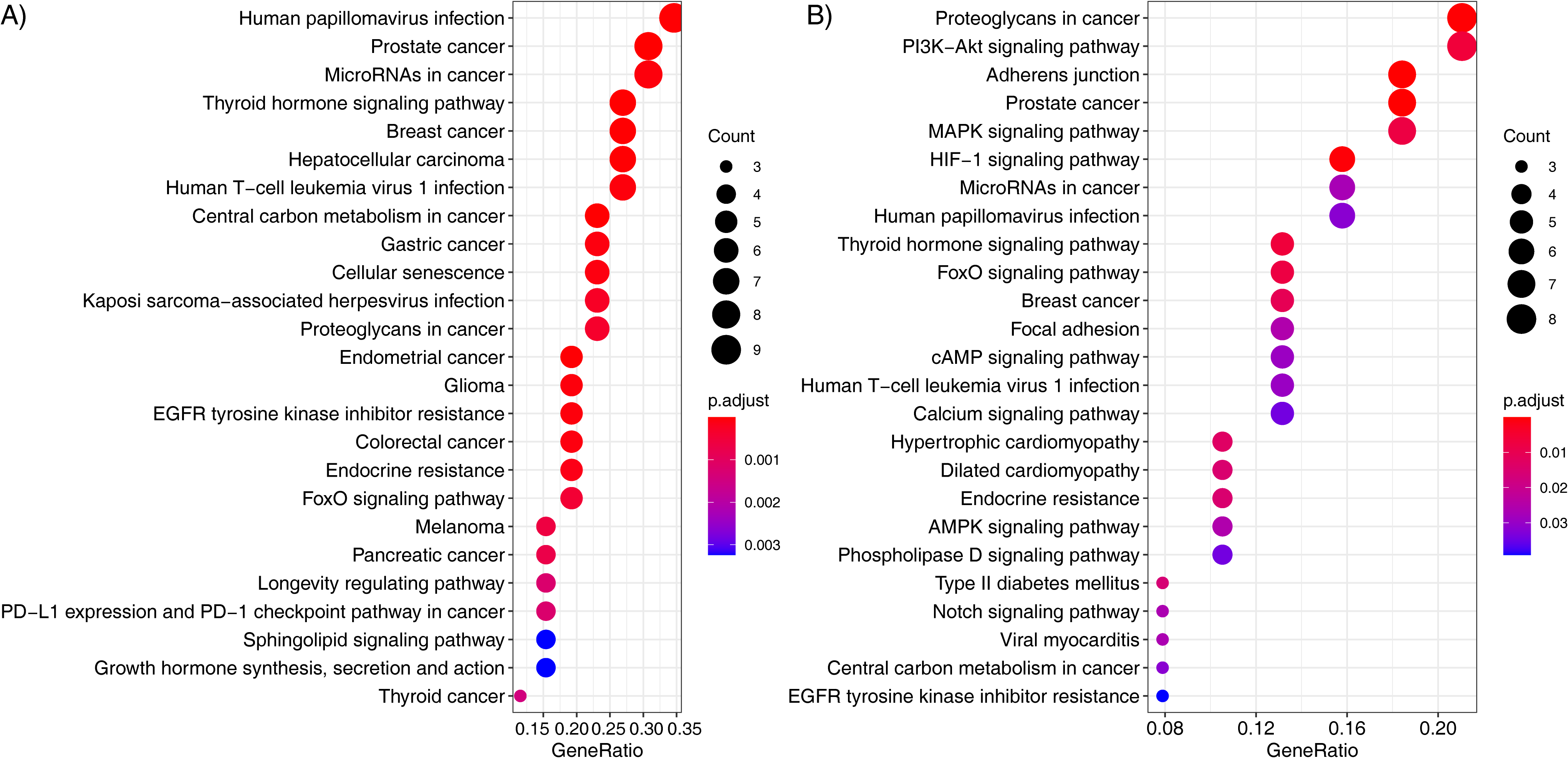


**Fig S7. The KEGG pathway enrichment analysis for the candidate driver genes of UCEC.** The x-axis represents gene ratio. The size of dot represents gene count. The color of the dot represents adjusted p-value. A) The results of top 30 candidate genes. B) The results of potential genes (genes in top 200 candidate gene list predicted by DriverRWH with both HumanNet and the STRINGv10 while not in tumor-specific drivers).

| Genes | Co-appeared count | | | Is_Specificity | Rank position | | | | | |
| --- | --- | --- | --- | --- | --- | --- | --- | --- | --- | --- |
|  | Endometrial | Cancer | Driver |  | MutsigCV | Dawnrank | Gravity | OncodriveFML | Subdyquency | MinNetRank |
| PTEN | 380 | 2597 | 35 | 1 | 1 | 7 | 168 | 2 | 2 | 1 |
| TP53 | 143 | 5942 | 55 | 1 | 5 | 3586 | 403 | 3 | 3 | 161 |
| PIK3CA | 39 | 576 | 13 | 1 | 4 | 36 | 673 | 22 | 1 | 2 |
| KRAS | 51 | 2538 | 95 | 1 | 9 | 66 | 8653 | 8583 | 10 | 35 |
| CTNNB1 | 112 | 2014 | 29 | 1 | 7 | 4 | 22 | 13663 | 5 | 860 |
| PIK3R1 | 3 | 97 | 6 | 1 | 2 | 3913 | 166 | 13309 | NA | 47 |
| DLG2 | 0 | 0 | 1 | 0 | 108 | 1290 | 4691 | 3173 | NA | 4468 |
| DICER1 | 6 | 150 | 5 | 1 | 275 | 11005 | 150 | 6636 | NA | 3720 |
| SMARCA4 | 1 | 123 | 5 | 0 | 1648 | 116 | 904 | 1094 | 102 | 98 |
| HSP90AB1 | 1 | 53 | 1 | 0 | 625 | 235 | 737 | 8947 | 100 | 8 |
| NEMF | 0 | 6 | 1 | 0 | NA | NA | NA | 9512 | NA | 5052 |
| TTN | 0 | 8 | 1 | 0 | 12 | 2992 | 1195 | 13175 | NA | 577 |
| SMC4 | 0 | 6 | 1 | 0 | 437 | 1061 | 822 | 5084 | NA | 1244 |
| DLG4 | 0 | 5 | 1 | 0 | 4277 | 4701 | 1476 | 10968 | NA | 5521 |
| POLR2A | 0 | 46 | 2 | 0 | 1290 | 9 | 236 | 3272 | 105 | 16 |
| SMARCA2 | 0 | 63 | 4 | 0 | 1079 | 169 | 751 | 11802 | 80 | 174 |
| CAD | 0 | 40 | 1 | 0 | 293 | 9548 | 1091 | 1470 | 120 | 357 |
| EP300 | 2 | 145 | 2 | 0 | 55 | 7996 | 17 | 10569 | NA | 3511 |
| KALRN | 0 | 5 | 1 | 0 | 277 | 3375 | 1004 | 12179 | 45 | 1041 |
| MAGI1 | 0 | 7 | 2 | 0 | 1536 | 7097 | 1564 | 10077 | NA | 5209 |
| GLI3 | 2 | 36 | 2 | 0 | 1046 | 11 | 3088 | 10354 | NA | 1172 |
| SMC1A | 0 | 16 | 1 | 0 | 686 | 1651 | 191 | 10399 | 93 | 114 |
| KMT2D | 0 | 18 | 1 | 1 | NA | NA | 3113 | 1 | NA | 3114 |
| NSUN2 | 0 | 9 | 1 | 0 | 1545 | 9473 | 1504 | 12456 | NA | 1479 |
| MAGI2 | 0 | 5 | 1 | 0 | 1473 | 1100 | 4403 | 2349 | NA | 5269 |
| TRIO | 0 | 12 | 3 | 0 | 282 | 5252 | 570 | 9821 | 34 | 898 |
| MAGI3 | 0 | 6 | 1 | 0 | 1042 | 8357 | 3723 | 7450 | NA | 5432 |
| MKI67 | 16 | 256 | 6 | 0 | 26 | 2 | 2216 | 1752 | NA | 3316 |
| DLG1 | 0 | 52 | 6 | 0 | 10581 | 4972 | 2266 | 2911 | 138 | 1642 |
| DROSHA | 1 | 77 | 3 | 0 | NA | NA | 1808 | 5131 | NA | 3614 |

**Table S3. Cociter mining analysis of top 30 UCEC candidate driver genes identified by DriverRWH (HumanNet).**


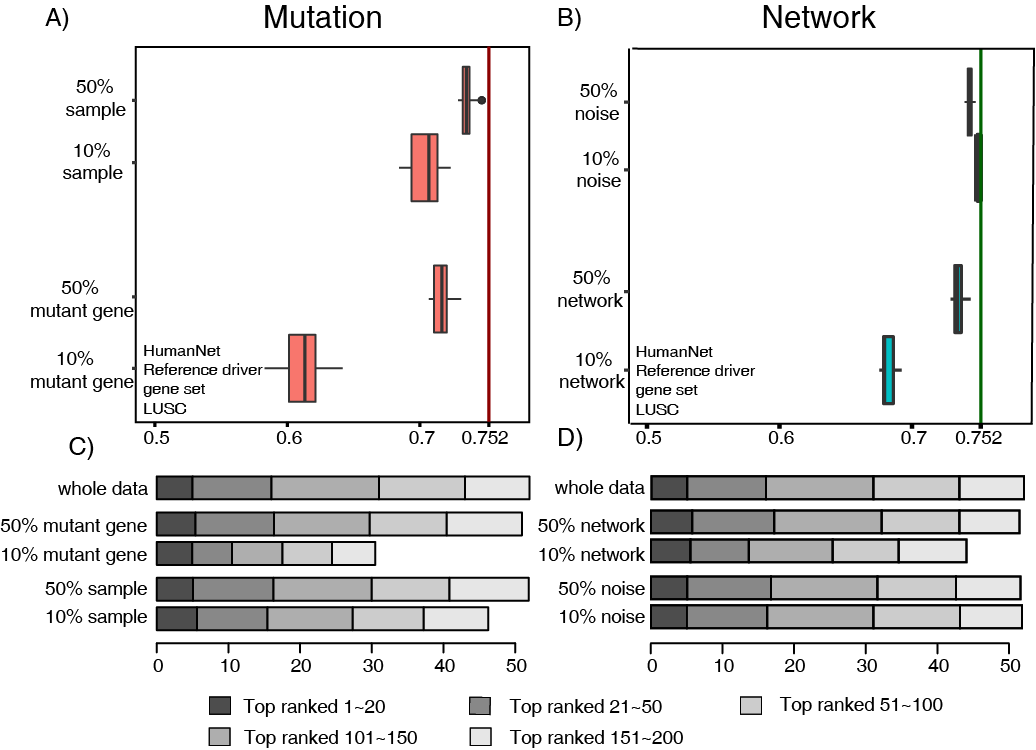


**Fig S8.** **Robustness of DriverRWH.** (A, B) Boxplots of the effects of different data perturbations on the performance of DriverRWH. The vertical lines represent the AUC scores by DriverRWH using all of the data. (C, D) Effects of different data perturbations on the performance of DriverRWH measured by the average cumulative number of known cancer genes recovered in the 20, 50, 100, 150, and 200 top-ranked candidate genes.
